# Synthesis of Some Benzofuran Derivatives Containing Pyrimidine Moiety as Potent Antimicrobial Agents

**Published:** 2018

**Authors:** Talavara Venkatesh, Yadav Dasharathrao Bodke, Muthipeedika Nibin Joy, Bhadrapura Lakkappa Dhananjaya, Sivaramakrishnan Venkataraman

**Affiliations:** a *Department of P.G. Studies and Research in Industrial Chemistry, Jnana Sahyadri, Kuvempu University Shankaraghatta, Shivamogga, Karnataka, India. *; b *Toxinology/Toxicology and Drug Discovery Unit, Centre for Emerging Technologies (CET), Jain University, Kanakpura Taluk, Ramanagara India.*; c *Cardiomyocyte Toxicity and Oncology Research Laboratory, Department of Bioinformatics, SCBT, Sastra University, Thanjavur, India.*

**Keywords:** Benzofuran chalcones, Hydroxy pyrimidines, Thiopyrimidines, Amino pyrimidines, Antimicrobial activity, Molecular docking studies

## Abstract

In this investigation, the synthesis of 2-substituted pyrimidines by the reaction of benzofuran chalcones (3a-d) with urea, thiourea and guanidine hydrochloride was reported. The structures of title compounds (4a-d), (5a-d) and (6a-d) were established on the basis of analytical and spectral data. The synthesized compounds were screened for antimicrobial activity and molecular docking studies. Some of the compounds displayed excellent antimicrobial activity. The molecular docking analysis revealed that compounds 5a and 5c with the lowest binding energy in comparison to others suggesting its potential as best inhibitor of GluN-6-P. Consequently, it is confirmed from the above analysis that the compounds 5a and 5c might serve as a useful backbone scaffold for rational design, adaptation and investigation of more active analogs as potential broad spectrum antimicrobial agents.

## Introduction

The cynosure towards pyrimidine based drug discovery is an emergent field in the heterocyclic chemistry. A significant feature of benzofuran chalcones is their capability to act as an intermediate for the synthesis of biologically active heterocyclic compound pyrimidine, which occupies a unique position in medicinal chemistry. The nitrogen and oxygen containing heterocyclic compounds have established great attention due to their wide range of pharmacological activities. Benzofuran chalcones are one of the most important classes of synthetic or biogenetic precursors with general manifestation in fruits, vegetables, spices and soya based foodstuffs, which have impressive array of biological and pharmacological activities (1). In addition, benzofuran chalcone derivatives are foremost class of organic compounds that occur in a number of natural products and its ring system is a common structural element that appears in a large number of important compounds (2), used in cosmetics and medicinal industry (3, 4). The condensed benzofuran with pyrimidine scaffold makes up the core structure of several biologically active compounds, notable among which are the antioxidant (5), anti-inflammatory, analgesic (6, 7), anti-plasmodial (8) and anti-amoebic agents (9). Furthermore, they also serve as inhibitors of 5-lipoxygenase and angiotensin II converting enzymes (10). From the recent literature, the role of pyrimidine entity is shown to attract essentiality in several biological processes, such as nucleoside antibiotics, multivitamin synthesis and functional activity maintenance of coenzymes (11). Nevertheless, much interest has been concentrated on the synthesis of pyrimidines possessing fungicidal, herbicidal, anti-depressant (12, 13), anti-tumor (14, 15), anti-infective (16), anti-convulsant (17), antimicrobial (18), anti-viral (19), anti-protozoal (20), anti-hypertensive (21), anti-helmintic (22), anti-tubercular (23), anticancer (24, 25) and anti-HIV (26) properties. It has also been reported as components of calcium-sensing receptor antagonists (27) and hypnotic (28, 29) drugs. Glucosamine-6-phosphate synthase (GlcN-6-P; EC 2.6.1.16) a key enzyme in cell wall biosynthesis catalyzes the first step in hexosamine biosynthesis, converting D-fructose 6-phosphate into D-glucosamine 6-phosphate using glutamine as the ammonia source (30-32). GlcN-6-P is a precursor of uridine diphospho-*N*-acetyl glucosamine from which other amino-sugar containing molecules were derived. One of these products, *N*-acetyl glucosamine, is an important constituent of the peptidoglycan layer of bacterial cell walls and fungal cell wall, chitin. Accordingly, GlcN-6-P serves as a promising target for antibacterial and antifungal drug discovery. In continuation of the research on benzofuran chalcone based pyrimidine derivatives (33-36), the present study was undertaken to investigate the antimicrobial property of the newly synthesized compounds using experimental and computational analysis. The experimental *in-vitro *analysis is being performed to report and evaluate newly synthesized pyrimidine derivatives as antimicrobial agents. Consequently, computational analysis of the potential antimicrobial compounds obtained from experimental analysis was subjected to molecular docking against GlcN-6-P to further investigate their possible binding interaction mode suggesting its potential role as target specific antimicrobial agents.

## Experimental

*Materials and Analytical methods*

All reactions were performed at room temperature. High speed stirring was carried out with magnetic force. All chemicals were purchased from Aldrich Chemical Company and solvents were used without further purification. Analytical thin-layer chromatography was performed with E. Merck silica gel GF254 glass plates. Visualization of the developed chromatogram was performed by UV light (254 nm). Column chromatography was performed on silica gel 90, 200–300 mesh. Melting points were determined with Shimadzu DS-50 thermal analyzer. The FT-IR spectra were obtained using KBr pellets on Shimadzu spectrometer. The ^1^H NMR and ^13^C NMR spectra were recorded on Bruker 400 and 100 MHz in DMSO-d_6_ as a solvent using tetramethylsilane (TMS) as internal standard respectively. LC-MS were obtained using C-18 column on Shimadzu, LCMS 2010A, Japan.

*General procedure for Synthesis of compounds (4a-d) and (5a-d)*

Equimolar quantity of benzofuran chalcones (1 mmol) (3a-d) were condensed with urea/thiourea in alcoholic KOH in a round bottom flask and the reaction mixture was continuously stirred for about 5-6 h at room temperature. The progress of the reaction was monitored by thin layer chromatography and spots were observed by iodine vapor and/or UV light. After completion of reaction, the reaction mixture was cooled, poured into crushed ice with constant stirring and neutralized using 10% NaHCO_3_. The precipitated product was filtered, dried and recrystallized using ethanol. 

*4-(1-Benzofuran-2-yl)-6-(thiophen-2-yl) pyrimidin-2-ol (4a)*

Yield: 79%. Brown solid (EtOH), mp 160-162 °C; IR (KBr, υ cm^-1^): 3464 (OH stretching), 1612 (C=N). ^1^HNMR (δ ppm): 7.32-7.30 (d, 2H, thiophene); 7.53-7.75 (m, 5H, ArH); 8.55 (s, 1H, pyrimidine); 8.78 (s, 1H, furan); 10.28 (s, 1H, OH).^13^CNMR (δ ppm): 100.12; 112.50; 112.86; 113.11; 116.96; 118.78; 123.24; 123.91; 127.33; 128.20; 130.38 (C-S), 145.29 (C=C); 151.84 (C-O); 153.78 (C-N); 155.84 (C=N); 159.43 (C-OH).MS, m/z: 295.30[M+1].

*4-(1-Benzofuran-2-yl)-6-(5-methylthiophen-2-yl)pyrimidin-2-ol (4b)*

Yield: 80%. Light brick red (EtOH), mp 154-156 °C. IR (KBr, υ cm^-1^): 3430 (OH stretching), 1610 (C=N). ^1^HNMR (δ ppm): 2.42 (s, 3H, CH_3_); 7.54-7.76 (m, 6H, ArH); 8.54 (s, 1H, pyrimidine); 8.75 (s, 1H, furan); 10.35 (s, 1H, OH). ^13^CNMR (δ ppm): 24.23 (CH_3_); 101.20; 108.20; 110.50; 111.20; 113.84; 115.60; 123.10; 124.76; 128.30; 129.80; 130.14 (C-S); 146.30 (C=C); 152.76 (C-O); 154.72 (C-N); 155.40 (C=N); 159.10 (C-OH). MS, m/z: 309.30[M+1].

*4-(5-Bromo-1-benzofuran-2-yl)-6-(thiophen-2-yl)pyrimidin-2-ol (4c)*

Yield: 86%. Brown solid (EtOH), mp 171-173 °C. IR (KBr, υ cm^-1^): 3430 (OH stretching), 1615 (C=N), 747(C-Br). ^1^HNMR (δ ppm): 7.30-7.28 (d, 1H, thiophene); 7.50-7.72 (m, 4H, ArH); 8.51 (s, 1H, pyrimidine); 8.76 (s, CH, furan); 10.22(s, 1H, OH). ^13^CNMR (δ ppm): 102.24; 110.30; 111.10; 111.80; 114.92; 116.80; 124.20; 125.86; 129.32; 130.10; 132.34 (C-S); 147.32 (C=C); 153.78 (C-O); 155.80 (C-N); 156.60 (C=N); 160.02 (C-OH). MS, m/z: 372[M+] and 374[M+2].

*4-(5-Bromo-1-benzofuran-2-yl)-6-(5-methylthiophen-2-yl)pyrimidin-2-ol (4d)*

Yield: 83%. Brick red (Et-OH), mp 170-172 °C. IR (KBr, υ cm^-1^): 3442 (OH stretching), 1614 (C=N), 677 (C-Br). ^1^HNMR (δ ppm): 2.43 (s, 3H, CH_3_); 7.53-7.76 (m, 5H, ArH,); 8.66 (s, 1H, pyrimidine); 8.74 (s, 1H, furan); 10.35(s, 1H, OH). ^13^CNMR (δ ppm): 23.20 (CH_3_); 100.00; 110.10; 111.20; 112.20; 114.80; 115.90; 123.25; 124.80; 129.22; 130.12; 132.30 (C-S); 147.40 (C=C); 153.74 (C-O); 154.89 (C-N); 156.50 (C=N); 160.12 (C-OH). MS, m/z: 386[M+] and 388[M+2]. 

*4-(1-Benzofuran-2-yl)-6-(thiophen-2-yl) pyrimidine-2-thiol (5a)*

Yield: 85%. Brown (EtOH), mp 158-160 °C. IR (KBr, υ cm^-1^): 2564 (SH), 1655 (C=N). ^1^HNMR (δ ppm): 7.33-7.35 (d, 2H, thiophene); 7.53-8.35 (m, 6H, ArH); 8.54 (s, 1H pyrimidine); 10.35 (s, 1H, SH). ^13^CNMR (δ ppm):100.50; 112.50; 112.86; 113.11; 116.96; 118.78; 123.24; 124.00; 127.33; 128.20; 130.38 (C-S); 145.29 (C=C); 151.54 (C-O); 153.78 (C-N); 155.84 (C=N); 179.43 (C-SH). MS, m/z: 311.30[M+1].

*4-(1-Benzofuran-2-yl)-6-(5-methylthiophen-2-yl)pyrimidine-2-thiol (5b)*

Yield: 76%. Brown (EtOH), mp 154-156 °C. IR (KBr, υ cm^-1^): 2560 (SH), 1598 (C=N). ^1^HNMR (δ ppm): 2.33 (s, 3H, CH_3_);7.51-8.34 (m, 7H, ArH); 8.45 (s, 1H, pyrimidine); 10.11 (s, 1H, SH).^13^CNMR (δ ppm): 21.86 (CH_3_); 101.30; 111.10; 111.84; 114.10; 117.90; 119.75; 124.34; 125.11; 128.44; 129.26; 131.40(C-S); 146.30 (C=C); 152.64 (C-O); 154.73 (C-N); 156.82 (C=N); 180.20 (C-SH). MS, m/z: 325.40[M+1].

*4-(5-Bromo-1-benzofuran-2-yl)-6-(thiophen-2-yl)pyrimidine-2-thiol (5c)*

Yield: 85%. Brownish yellow (EtOH), mp 157-159 °C. IR (KBr, υ cm^-1^): 2558 (SH), 1600 (C=N), 746 (Ar-Br). ^1^HNMR (δ ppm): 7.30- 7.28 (d, 2H thiophene); 7.52-8.33 (m, 5H, ArH); 8.48 (s, 1H pyrimidine); 10.24 (s, 1H, SH). ^13^CNMR (δ ppm):100.20; 112.15; 113.88; 116.18; 118.80; 120.70; 123.30; 123.18; 127.46; 128.36; 130.50 (C-S); 145.40 (C=C); 151.74 (C-O); 154.83 (C-N); 156.92 (C=N); 179.50 (C-SH). MS, m/z: 388[M+] and 390[M+2].

*4-(5-Bromo-1-benzofuran-2-yl)-6-(5-methylthiophen-2-yl)pyrimidine-2-thiol (5d)*

Yield: 75%. Light Brown (EtOH), mp 162-163 °C. IR (KBr, υ cm^-1^): 2556 (SH), 1602 (C=N), 782 (Ar-Br).^1^HNMR (δ ppm): 2.42 (s, 3H, CH_3_); 7.54-8.36 (m, 6H, ArH); 8.42 (s, 1H pyrimidine); 10.28 (s, 1H, SH). ^13^CNMR (δ ppm): 24.42 (CH_3_); 102.35; 112.15; 112.88; 115.18; 118.80; 120.70; 121.50; 125.30; 126.18; 129.46; 133.10 (C-S); 148.42 (C=C); 154.14 (C-O); 155.00 (C-N); 156.60 (C=N); 179.46 (C-SH). MS, m/z: 402[M+] and 404[M+2].

*General procedure for Synthesis of 4-(5-substituted-1-benzofuran-2-yl)-6-(5-substituted thiophen-2-yl)pyrimidin-2-amine (6a-d)*

Equimolar quantity of benzofuran chalcones (1 mmol) (3a-d) was condensed with guanidine hydrochloride (1 mmol) in alcoholic KOH in a round bottom flask and reaction mixture was continuously stirred for about 5-6 h at room temperature. The progress of the reaction was monitored by thin layer chromatography and spots were observed by iodine vapor and/or UV light. After completion of reaction, the reaction mixture was cooled, poured into crushed ice with constant stirring and neutralized using 10% NaHCO_3_. The precipitated product was filtered dried and recrystallized using ethanol. 

*4-(1-Benzofuran-2-yl)-6-(thiophen-2-yl)pyrimidin-2-amine (6a*)

Yield: 83%. Dark Brown (EtOH), mp 168-170 °C. IR (KBr, υ cm^-1^): 3318 (NH_2_), 1625 (C=N). ^1^HNMR (δ ppm): 7.34-7.30 (m, 4H, ArH,); 7.560-7.543 (d, 2H, thiophene); 7.60 (s, 1H, furan); 7.66 (s, 1H, pyrimidine); 8.58 (s, 2H, NH_2_). ^13^CNMR (δ ppm): 101.20; 108.50; 110.37; 119.22; 120.06; 123.70; 126.09; 126.98; 129.17; 131.93; 134.98 (C-S), 145.82 (C=C);151.60 (C-O); 153.80 (C-N); 155.80 (C=N); 164.24 (C-NH_2_). MS, m/z: 294.30[M+1].

*4-(1-Benzofuran-2-yl)-6-(5-methylthiophen-2-yl)pyrimidin-2-amine (6b)*

Yield: 79%. Brown (EtOH), mp: 163-165 °C. IR (KBr, υ cm^-1^): 3315 (NH_2_), 1620 (C=N). ^1^HNMR (δ ppm): 2.40 (s, 3H, CH_3_); 7.33-7.29 (m, 6H, ArH); 7.59 (s, 1H, furan); 7.64 (s, 1H, pyrimidine); 8.54 (s, 2H, NH_2_). ^13^CNMR (δ ppm): 22.20 (CH_3_); 100.10; 107.51; 111.17; 118.11; 121.16; 124.60; 127.19; 127.96; 130.18; 131.94; 135.88 (C-S), 146.00 (C=C); 151.66 (C-O); 154.82 (C-N); 156.68 (C=N); 165.00 (C-NH_2_). MS, m/z: 308.30[M+1]*.*

*4-(5-Bromo-1-benzofuran-2-yl)-6-(thiophen-2-yl)pyrimidin-2-amine (6c)*

Yield: 84%. Light brown (EtOH), mp 160-162 °C. IR (KBr, υ cm^-1^): 3320 (NH_2_), 1612 (C=N), 666 (Ar-Br). ^1^HNMR (δ ppm):7.35-7.31 (m, 4H, ArH); 7.561-7.543 (d, 2H, thiophene); 7.51 (s, 1H, furan); 7.62 (s, 1H pyrimidine); 9.12 (s, 2H, NH_2_). ^13^CNMR (δ ppm): 102.30; 109.01; 112.17; 119.10; 122.10; 125.00; 128.29; 128.96; 131.12; 132.84; 136.78 (C-S), 145.10 (C=C); 151.23 (C-O); 155.22 (C-N); 157.60 (C=N); 165.12 (C-NH_2_). MS, m/z: 371[M+] and 373[M+2].

*4-(5-Bromo-1-benzofuran-2-yl)-6-(5-methylthiophen-2-yl)pyrimidin-2-amine (6d)*

Yield: 77%. Brown (EtOH), mp 167-169 °C, IR (KBr, υ cm^-1^): 3318 (NH_2_), 1623 (C=N), 688 (Ar-Br). ^1^HNMR (δ ppm): 2.42 (s, 3H, CH_3_); 7.31-7.28 (m, 5H, ArH); 7.56 (s, 1H, furan); 7.60 (s, 1H, pyrimidine); 8.94 (s, 2H, NH_2_). ^13^CNMR (δ ppm): 23.21 (CH_3_); 101.10; 106.01; 112.07; 119.10; 122.26; 123.68; 128.19; 128.86; 131.08; 132.04; 136.18 (C-S), 145.10 (C=C); 150.96 (C-O); 155.12 (C-N); 156.68 (C=N); 165.00 (C-NH_2_). MS, m/z: 385[M+] and 387[M+2].

**Table 1:** Characterization data of synthesized compounds (4a-d), (5a-d) and (6a-d).

**Compd**	**R**	**R**_1_	**Yield (%)**	**Mol.wt**
4a	H	H	79	294.32
4b	H	CH_3_	80	308.35
4c	Br	H	86	373.22
4d	Br	CH_3_	83	387.25
5a	H	H	85	310.93
5b	H	CH_3_	76	324.41
5c	Br	H	85	389.28
5d	Br	CH_3_	75	403.31
6a	H	H	83	293.34
6b	H	CH_3_	79	307.36
6c	Br	H	84	372.23
6d	Br	CH_3_	79	386.26

**Table 2:** Anti-bacterial activity data of synthesized compounds (4a-d), (5a-d) and (6a-d).

**Compounds**	**Zone of inhibition (in mm)**					
	***B. subtilis***		***E. coli***		***P. aeruginosa***	
	**100 (mg)**	**50 (mg)**	**100 (mg)**	**50 (mg)**	**100 (mg)**	**50 (mg)**
4a	16.87 ± 0.78	6.78 ± 0.71	16.74 ± 0.45	6.57 ± 0.57	15.78 ± 0.42	6.15 ± 0.56
4b	18.80 ± 0.44	11.27 ± 0.81	18.21 ± 0.23	7.15 ± 0.54	17.28 ± 0.45	6.71 ± 0.51
4c	16.72 ± 0.51	7.78 ± 0.71	15.87 ± 0.27	6.45 ± 0.52	14.71 ± 0.43	6.72 ± 0.57
4d	19.20 ± 0.44	9.72 ± 0.57	18.82 ± 0.51	7.54 ± 0.24	17.87 ± 0.51	7.84 ± 0.57
5a	19.74 ± 0.27	9.72 ± 0.57	18.87 ± 0.51	7.54 ± 0.24	17.87 ± 0.51	7.84 ± 0.57
5b	17.41 ± 0.24	8.78 ± 0.41	17.15 ± 0.57	8.25 ± 0.48	16.27 ± 0.51	6.45 ± 0.51
5c	19.72 ± 0.53	10.62 ± 0.48	17.54 ± 0.31	7.24 ± 0.57	18.72 ± 0.51	7.25 ± 0.35
5d	19.60 ± 0.42	9.24 ± 0.57	18.81 ± 0.45	6.18 ± 0.57	17.74 ± 0.52	7.71 ± 0.68
6a	15.25 ± 0.05	7.12 ± 0.21	14.41 ± 0.54	6.13 ± 0.45	13.21 ± 0.71	6.15 ± 0.56
6b	16. 23 ± 0.47	6.41 ± 0.28	15.74 ± 0.81	0	14.84 ± 0.28	0
6c	15.23 ± 0.57	0	14.74 ± 0.23	0	13.74 ± 0.75	0
6d	14.41 ± 0.54	8.24 ± 0.52	13.21 ± 0.71	6.18 ± 0.57	14.74 ± 0.48	6.71 ± 0.58
Streptomycin	21.71 ± 0.41		20.45 ± 0.38		19.78 ± 0.48	
Control	0		0		0	

Each value is the mean of three replicate determinations ± standard deviation.

B.S: *Bacillus subtilis*; P.A: *Pseudomonas aeruginosa*; E.C: *Escherichia coli*.

**Table 3:** Antifungal activity data of synthesized compounds (4a-d), (5a-d) and (6a-d).

**Compounds**	**Zone of inhibition (mm)**					
	***A. alternata***		***A. niger***		***C.albicans***	
	**100 (mg)**	**50 (mg)**	**100 (mg)**	**50 (mg)**	**100 (mg)**	**50 (mg)**
4a	17.28 ± 0.57	6.15 ± 0.62	16.71 ± 0.87	6.78 ± 0.58	17.17 ± 0.54	6.17 ± 0.85
4b	17.49 ± 0.57	6.74 ± 0.58	17.81 ± 0.54	6.78 ± 0.71	16.54 ± 0.51	6.87 ± 0.45
4c	14.48 ± 0.54	12.45± 0.3	16.87 ± 0.45	6.21 ± 0.41	16.72 ± 0.37	6.58 ± 0.71
4d	19.78 ± 0.47	8.74 ± 0.71	18.87 ± 0.27	7.74 ± 0.48	18.78 ± 0.71	7.42 ± 0.59
5a	20.87 ± 0.57	9.27 ± 0.67	19.71 ± 0.41	8.27 ± 0.71	18.47 ± 0.47	8.57 ± 0.67
5b	17.78 ± 0.42	6.78 ± 0.81	16.74 ± 0.57	10.45±0.57	15.87 ± 0.58	11.21±0.27
5c	20.78 ± 0.57	8.27 ± 0.68	19.41 ± 0.28	8.27 ± 0.87	18.28 ± 0.35	7.47 ± 0.89
5d	19.38 ± 0.37	8.45 ± 0.37	18.43 ± 0.51	7.46 ± 0.81	16.45 ± 0.34	6.75 ± 0.27
6a	18.24 ± 0.45	7.17 ± 0.62	17.59 ± 0.42	6.37 ± 0.64	15.45 ± 0.43	6.87 ± 0.41
6b	15.07 ± 0.43	0	16.78 ± 0.51	0	15.35 ± 0.41	0
6c	16. 45 ± 0.47	6.87 ± 0.72	17.54 ± 0.45	6.15 ± 0.62	18.74 ± 0.53	7.84 ± 0.81
6d	14.78 ± 0.54	6.75 ± 0.47	16. 41 ± 0.23	0	16.57 ± 0. 78	0
Fluconazole	22. 87 ± 0.43		21.57 ± 0.35		20.78 ± 0.47	
Control	0		0		0	

Each value is the mean of three replicate determinations ± standard deviation.

C.A:* Candida albicans*; A.N:* Aspergillus niger*; A.A:* Alternaria alternate.*

**Table 4:** MIC value of synthesized compounds (4a-d), (5a-d) and (6a-d).

**Compound**	**Minimum inhibitory concentration µg/mL ± SD**								
	***B. subtilis***	***E. coli***	***P. aeruginosa***	***A. alternata***			***A. niger***		***C. albicans***
4a	15.32 ± 0.28	13.14 ± 0.41	14.25 ± 0.51		10.32± 0.27	8.27± 0.41		9.45 ± 0.28	
4b	13.10 ± 0.40	15.12 ± 0.26	10.11 ± 0.63		9.42± 0.30	7.23 ± 0.40		9.54 ± 0.52	
4c	11.22 ± 0.27	8.34 ± 0.43	10.27 ± 0.37		12.21 ± 0.41	11.77 ± 0.43		10.25 ± 0.26	
5a	18.45 ± 0.23	16.27 ± 0.41	12.34 ± 0.32		15.15 ± 0.48	13.81 ± 0.36		14. 57 ± 0.37	
5b	16.24± 0.26	12.47 ± 0.46	11.47 ± 0.28		12.61 ± 0.51	11.14 ± 0.41		10.92 ± 0.37	
5c	17.32 ± 0.24	15.21 ± 0.37	16.20 ± 0.45		14.32± 0.42	10.41± 0.47		11. 27± 0.45	
5d	15.25 ± 0.62	13.87 ± 0.45	14.15 ± 0.21		11.32 ± 0.21	10.25 ± 0.38		12.12 ± 0.51	
6a	10.32± 0.37	9.87± 0.51	6.64± 0.27		5.32± 0.42	3.21± 0.45		4.21± 0.51	
6b	6.32± 0.41	5.47± 0.51	4.32 ±0.57		4.57 ± 0.54	1.84 ± 0.62		3.27± 0.54	
Streptomycin	19.37 ± 0.23	17.25 ± 0.35	18.44 ± 0.45		-	-		-	
Fluconazole	-	-	-		18.54 ± 0.32	16.72 ± 0.27		17.45 ± 0.21	

Each value is the mean of three replicate determinations ± standard deviation.

B.s:* Bacillus subtilis*; P.a:* Pseudomonas aeruginosa*; E.c:* Escherichia coli*; C.a:* Candida *

**Table 5:** Molecular docking results of compounds (4a-d), (5a-d) and (6a-d) with inhibitory activity against G6P

**Molecule **	**Binding energy** **(Kcal mol** ^-1^ **)**		**RM SD**	**No. of hydrogen bonds**		**Bonding residues**	**Bond length** **(A°)**
4a	-5.26	0.43			1	Cys1; Trp74	2.8; 3.4
4b	-5.13	0.36			2	Cys1; Trp74	2.9; 3.3
4c	-4.82	0.42			2	Cys1; Trp74	2.7; 3.4
5a	-5.77	0.34			2	Cys1; Trp74	2.9; 3.4
5c	-5.29	0.38			2	Cys1; Trp74	2.6; 3.5
5d	-5.25	0.32			2	Trp74	3.2
6a	-3.5	0.38			2	Cys1; Trp74	3.0 and 3.3
Streptomycin	-5.99	0.35			3	Trp74; Ala75; Thr76	2.8, 2.2 and 1.9
Fluconazole	-5.47	0.37			3	Trp74; His97; Gly99	3.1, 2.7 and 2.9

**Table 6:** ADME/Toxicity values of the synthesized compounds (4a-d), (5a-d) and (6a-d) considered for this study

**Molecule**	a **MW**	b **logp**	c **HBA**	d **HBD**	e **BBB**	f **Caco2**	g **HIA**
4a	294.32	1.667	4	0	2.20	31.9876	96.89893
4b	308.35	1.518	4	0	0.26	23.2461	96.81469
4c	373.22	2.209	4	0	0.51	27.5327	96.60848
5a	310.93	2.292	3	0	2.93	54.9743	99.74191
5c	389.28	2.834	3	0	3.36	53.7249	98.54442
5d	403.31	2.685	3	0	2.87	54.7978	98.37033
6a	293.34	1.256	4	0	1.94	22.3857	97.79064
Streptomycin	581.57	-5.988	19	12	0.04	9.38822	0
Fluconazole	306.27	-0.958	7	1	0.24	17.2331	95.58981

a MW: Molecular Weight;

b logP: Partition coefficient of Octonol/water;

c HBA: Hydrogen bond acceptor;

d HBD: Hydrogen bond donor;

e BBB: Blood brain barrier;

f Caco2: Caco-2 cell permeability indicative of gut-blood barrier;

g HIA: Human Intestinal absorption.

**Scheme 1:**
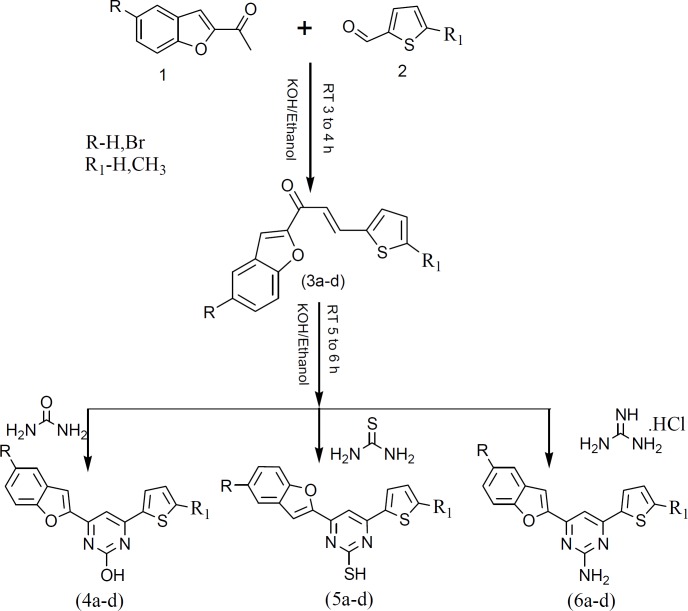
Synthesis of benzofuran pyrimidine derivatives (4a-d), (5a-d) and (6a-d).

**Figure 1:**
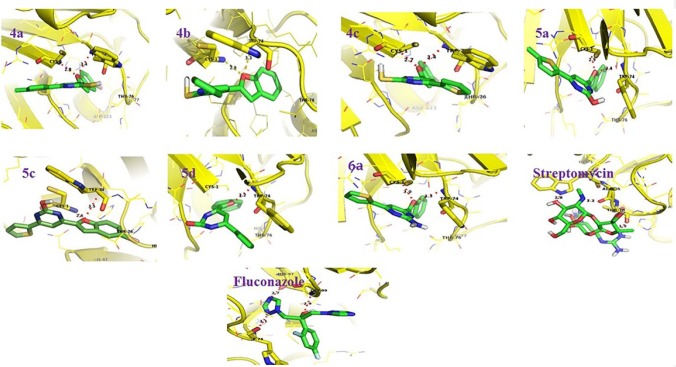
G6P-Ligand interactions as visualized using pymol (Version 1.3). The protein molecule is represented as ribbons (yellow color). The interacting residues (yellow color) and the ligands (green color) are represented as sticks (green color). The hydrogen bonds are represented as dotted lines (red color

*In-vitro evaluation of anti-microbial activity;*

All synthesized compounds were evaluated *in-vitro *for their antibacterial activity against gram positive bacteria *Bacillus subtilis *(MTCC 1134), gram negative bacteria *Escherichia coli *(MTCC 1559) and *Pseudomonas aeruginosa *(MTCC 1034). The antifungal activity against *Alternaria alternate *(MTCC 3793)*, Aspergillus niger *(MTCC 4325) and *Candida albicans* (MTCC 1637). Agar well diffusion technique was used for the determination of preliminary antibacterial and antifungal activities (37). Streptomycin and fluconazole were used as reference drugs for comparison. The tested compounds were dissolved in DMSO to get a concentration of 100% and 50%. The samples were loaded into wells of agar plates directly. Plates inoculated with the bacteria were incubated at 37 °C for 24 h and the fungal culture was incubated at 25 °C for 72 h. All determinations were done in triplicates. The results were recorded for each tested compound as average diameter of inhibition zones around the well in mm. The minimum inhibitory concentration (MIC) was performed by serial broth-dilution method (National Committee for Clinical Laboratory Standards, 1982). 

*Computational*

*In-silico molecular docking studies;*

*Ligand Preparation*

The structure of potential antimicrobial derivatives (Compound 4a, 4b, 4c, 5a, 5c, 5d and 6a) and known GluN-6-P inhibitors (Streptomycin and Fluconazole) were drawn using ChemDraw (Version 10). All the ligands were 3D optimized using PRODRG server (38). All the ligands were prepared for molecular docking by merging the non-polar hydrogens, assigning of Gastegier charges, and saving them in PDBQT file format using Auto Dock Tools (ADT) 1.5.6.

*Protein preparation*

The X-ray crystal structure of GluN-6-P (PDB ID: 1XFF) with a resolution of 1.81 Å was obtained from the Protein Data Bank (http://www.rcsb.org/pdb). All the heteroatoms and water molecules were removed. Polar hydrogens were removed and Gasteiger charges were assigned after merging of non-polar hydrogen atoms. After such preprocessing procedure of the crystal structure, it was saved in PDBQT file format using ADT.

*Molecular docking*

Automated docking was performed with the Auto Dock 4 (Scripps Research Institute, USA) using an empirical free energy function and Larmarckian Genetic Algorithm, with an initial population of 250 randomly placed individuals, a maximum number of 10^6^ energy evaluations, a mutation rate of 0.02, and a crossover rate of 0.80. The grid map was centered at the active site binding pocket of the protein by AutoGrid 4 involving Cys 1, Trp 74, Thr 76, His 77, Gly 99, and Asp 123 as previously reported by other groups (39-41). One-hundred independent docking runs were performed for each ligand. Results differing by < 2.0 A^°^ in positional root-mean-square deviation (RMSD) were clustered together and represented by the result with the most favorable free energy of binding. All torsions were allowed to rotate during docking.

*Validation of docking*

To ensure that the binding possess obtained from the docking studies are likely to represent authentic binding interactions, the removal and re-docking of the potential ligands/reference ligands (streptomycin/fluconazole) against GluN-6-Pwere performed more than 10 runs. The RMSD of the overlapping structures and reoccurrence of the similar binding pose was ascertained to confirm the docking procedure effectiveness.

*Absorption-Distribution-Metabolism-Excretion and toxicity (ADME/Tox)*

The ADME properties of all the ligands were calculated by using the QikProp program (Version 3.4). Qikprop predicts physically significant descriptors and pharmaceutically relevant properties of chemical molecules. Ligprep minimized ligands were given as a source in Qikprop. More than 40 chemical and biological descriptors with relevance to drug-likeliness were analyzed for all the compounds considered in this study (42).

## Results and Discussion

*Experimental analysis*

Synthesis of the target compounds were achieved according to the reactions illustrated in the Scheme 1.

Chalcone intermediates were obtained by Aldol condensation of corresponding 2-acetyl benzofuran (1) with different thiophene carbaldehydes (2) according to the reported procedure (43). The condensed heterocyclic compounds (4a-d) and (5a-d) were synthesized by treating (2E)-1-(5-substituted-1-benzofuran-2-yl)-3-(thiophen-2-yl)prop-2-en-1-one (3a-d) with urea and thiourea respectively in ethanoic KOH to form 4-(5-substituted-1-benzofuran-2-yl)-6-(thiophen-2-yl) pyrimidin-2-ol (4a-d) and 4-(5-substituted-1-benzofuran-2-yl)-6-(thiophen-2-yl) pyrimidin-2-thiol (5a-d) analogs. The 4-(5-substituted-1-benzofuran-2-yl)-6-(thiophen-2-yl) pyrimidin-2-amine (6a-d) analogs were obtained by the reaction of (2E)-1-(5-substituted-1-benzofuran-2-yl)-3-(thiophen-2-yl)prop-2-en-1-one (3a-d) with guanidine hydrochloride in the presence of ethanoic KOH. 

The structure of desired pyrimidine analogs (4a-d), (5a-d) and (6a-d) were confirmed using IR, NMR and Mass spectral data. The IR spectrum of compound 4a exhibited a broad absorption band at 3464 cm^-1 ^which corresponds to hydroxyl group. Another absorption band in the region of 1612 cm^-1 ^corresponds to C=N stretching vibration. The ^1^HNMR spectrum of compound 4a exhibited a broad singlet at δ 11.25 ppm due to hydroxyl proton and multiplet between δ 7.30-8.78 ppm for aromatic protons. Further, ^13^CNMR spectrum of compounds 4a displayed the signals at required positions. The mass spectrum of the compound 4a showed molecular ion peak at m/z 295.30[M+1] which corresponds to molecular weight of the compound. The physical and analytical data of the newly synthesized compounds (4a-d), (5a-d) and (6a-d) are tabulated in Table 1.

*Biological activity*

Though, there are several synthetic drugs in the market, the bacterial mutations assemble their resistance. To overcome this challenge, there is a crucial need to discover the new and specific synthetic molecules against microbes. In view of this, all the synthesized compounds in the present investigation (4a-d), (5a-d) and (6a-d) were evaluated for their antimicrobial activity by a primary screening analysis. All the synthesized compounds exhibited significant antimicrobial activity against bacterial strains *Bacillus subtilis*. The results of antibacterial and antifungal activity at two different concentrations (50 mg and 100 mg) have been depicted in Tables 2 and 3.

Further, the compounds which showed good antimicrobial activity in primary screening were assessed to determine minimum inhibitory concentration by broth dilution method and results were illustrated in Table 4.

Recent literature suggests that the structural and electronic parameters of synthesized compounds may have better impact on changing the efficacy of antimicrobial activity (44). A primary study shows that all the compounds displayed a varied degree of MIC (18.45 ± 0.23 to 5.47 ± 0.51 µg/mL) against all the tested bacterial strains. The compound 5a and 5c exhibited excellent antibacterial activity against bacterial strain *Bacillus subtilis* with MIC value 18.45 ± 0.23 and 17.32 ± 0.24 µg/mL. The compound 5b exhibit good antibacterial activity against all the tested bacterial strains with MIC value in the range of 16.24 ± 0.26 to 11.47 ± 0.28 µg/mL. Compounds 4a, 5d and 4b showed moderate effect against tested pathogenswith MIC value in the range of 15.32 ± 0.28 to 10.11 ± 0.63 µg/mL. The compounds 4c and 6a showed considerable activity against all the tested bacterial strains with MIC value in the range of 11.22 ± 0.27 to 6.64 ± 0.27 µg/mL. Nevertheless, the remaining compounds showed negligible antibacterial activity against all the tested strains.

 The antifungal action of newly synthesized compounds also indicate that compounds 4a, 4b, 4c, 5a, 5c, 5d and 6a exhibited excellent antifungal activity against all tested pathogens with MIC value in the range of 15.15 ± 0.48 to 3.21 ± 0.45 µg/mL. The least MIC values were found in the remaining compounds as tabulated in Table 4. All the experimental analysis showed that presence of benzofuran compound incorporated with thiol and phenolic group moiety increases the antimicrobial activity of chosen compounds*.* Henceforth only these potential antimicrobial derivatives (compounds 4a, 4b, 4c, 5a, 5c, 5d and 6a) and known GluN-6-P inhibitors (Streptomycin and Fluconazole) were further considered for computational analysis.

*Computational analysis*

 GluN-6-P is the first rate limiting enzyme responsible for the synthesis of N-acetyl glucosamine that is needed for cell wall polypeptide biosynthesis in bacteria and further for chitin synthesis in fungus. The present study involving *in-silico* analysis was undertaken to identify whether the molecular docking of benzofuran containing pyrimidine derivatives (4a, 4b, 4c, 5a, 5c, 5d and 6a) with GluN-6-P provides any correlation with their *in-vitro* antibacterial activity. Computationally the molecular docking investigations revealed the similar binding energy values of benzofuran derivatives in comparison to reference compounds like streptomycin and fluconazole (Table 5) suggestive of their potential antimicrobial activity based on lowest binding energy to GluN-6-P.

 Additionally, the RMSD value was obtained for docking and re-docking of potential antimicrobial compounds and standard drugs streptomycin/Fluconazole. The molecular docking analysis revealed that, compounds 5a and 5c with the lowest binding energy in comparison to others suggesting its potential as best potential inhibitor of GluN-6-P (Table 5).

 Most of the benzofuran derivatives were found to be interacting via hydrogen bond interactions with key active site residues Cys 1 and TRP 74 (Figure 1) of GluN-6-P suggestive of its inhibitory potential.

 Furthermore, ADME/Tox analysis of the benzofuran containing pyrimidine derivatives was performed to check for the drug likeliness property and human intestinal absorption (HIA). This analysis also revealed that, compound 5a and 5c was nontoxic and has the best human intestinal absorption in comparison to all other ligands (Table 6).

 Nevertheless, streptomycin had poor HIA in comparison to benzofuran derivatives. All of the above findings demonstrated that computational analysis was in good agreement with *in-vitro* observations.

## Conclusion

 In present work, biologically active benzofuran compounds containing pyrimidine ring were synthesized from benzofuran chalcones having high chemical reactivity and diverse synthetic applications. From antimicrobial activity results, it was found that the presence of hydroxyl, thiol, and amino groups in the pyrimidine ring displayed promising antimicrobial activity. Additionally, the molecular docking analysis revealed that, compounds 5a and 5c with the lowest binding energy were suggestive of the highest binding affinity in comparison to other compounds. Furthermore, computational analysis also revealed favorable ADME/Tox features of compounds 5a and 5c. Among the compounds studied, only 5a and 5c showed significant human intestinal absorption. From all the experimental and computational analysis it may be concluded that benzofuran compounds fused with pyrimidine ring showed significant broad spectrum of antimicrobial activity and had high affinity against GluN-6-P. Henceforth it can serve as new building blocks for synthesis and design of broad spectrum antimicrobial compounds.
